# Global insights into pediatric ischemic stroke: a bibliometric and visualization analysis

**DOI:** 10.3389/fmed.2026.1708673

**Published:** 2026-03-16

**Authors:** Yangyang Zhou, Xiaoye Huang, Kai Cai, Aguo Li, Dongmei Huang, Wenyue Si, Kaiying Yang

**Affiliations:** 1 Department of Neurosurgery, Shenzhen Children's Hospital, Shenzhen, China; 2 School of Pediatrics, Guangzhou Medical University, Guangzhou, China; 3 The Second Clinical College, Guangzhou Medical University, Guangzhou, China; 4 Department of Thoracic Surgery Guangzhou Women and Children’s Medical Center, Guangzhou Medical University, Guangzhou, China; 5 Department of Science Research and Education Management, Guangzhou Women and Children’s Medical Center, National Children’s Medical Center for South Central Region, Guangzhou Medical University, Guangzhou, China; 6 Department of Dermatology, Zhujiang Hospital of Southern Medical University, Guangzhou, China; 7 Department of Pediatric Surgery, Guangzhou Women and Children's Medical Center, National Children's Medical Center for South Central Region, Guangzhou Medical University, Guangzhou, China

**Keywords:** bibliometric analysis, children, ischemic stroke, mechanical thrombolysis, pediatric

## Abstract

**Background:**

Ischemic stroke (IS) is a cardiovascular disorder resulting from the obstruction of cerebral blood vessels, which significantly affects children’s long-term quality of life due to chronic neurological sequelae. This study aims to provide a comprehensive bibliometric analysis of research trends and hotspots in pediatric IS.

**Methods:**

Relevant publications on pediatric IS from 2000 to 2024 were retrieved from the Web of Science Core Collection (WOSCC) and PubMed database. Bibliometric analyses and visualizations were performed using VOSviewer, CiteSpace, and the Bibliometrix R package.

**Results:**

A steadily increasing publication trend has been observed since 2000. The most cited study was published in The Lancet by Feigin et al. in 2014. Pediatric Neurology had the highest number of publications, whereas Stroke ranked first in citations and H-index. The United States led in both publication volume and collaborative networks, while the Hospital for Sick Children in Canada stood out with the highest number of publications and citations among institutions. Gabrielle deVeber was the most prolific and influential author in terms of publications count and H-index. Current research hotspots mainly focus on etiological investigations, clinical interventions, and multidisciplinary collaborative approaches in the management of pediatric IS.

**Conclusion:**

This study provides a bibliometric analysis to systematically explore research trends and development in pediatric IS. Enhanced international and institutional collaborations are needed to promote the equitable dissemination of scientific knowledge and resources in this field.

## 1 Introduction

Ischemic stroke (IS) is characterized by cerebral ischemia, hypoxia, and neurological dysfunction resulting from the obstruction of cerebral blood vessels, accounting for approximately 86.8% of all strokes (1). In 2021, the reported prevalence of IS was 92.4 per 100,000 according to epidemiological data (2). Compared to adults, IS is relatively uncommon in children, with an incidence of approximately 2.09 per 100,000, but it occurs more frequently in neonates (3). In contrast to adults, pediatric IS is etiologically distinct, with vasculopathies recognized as an important underlying cause in children (4). Identified risk factors for pediatric IS include congenital heart disease, a genetic predisposition to thrombosis, infections, and trauma (5, 6). According to data from the Canadian Pediatric Ischemic Stroke Registry, the mortality rate for IS in children is low (7). Nevertheless, up to 60% of neonates and 70% of older children may develop chronic neurological sequelae, substantially affecting their long-term quality of life (7, 8).

Bibliometric analysis has emerged as a valuable method for examining research trends and development within a specific scientific field (9). By quantitatively assessing citations, publication sources, and keywords, bibliometric analyses can identify research hotspots, trace the historical evolution of a topic, and forecast future directions (9). The Web of Science Core Collection (WOSCC), which encompasses various document types, is a widely recognized and powerful resource for citation analysis, journal ranking, and impact tracking indicators in bibliometric studies (10).

In this study, we performed a comprehensive bibliometric analysis to evaluate research on pediatric IS from multiple perspectives, including journals, countries, institutions, authors, and keywords. By systematically mapping current research trends in pediatric IS, our findings aim to enhance understanding of this field and provide a foundation for future collaborative efforts and clinical applications.

## 2 Materials and methods

### 2.1 Data sources and search strategies

**Figure 1:**
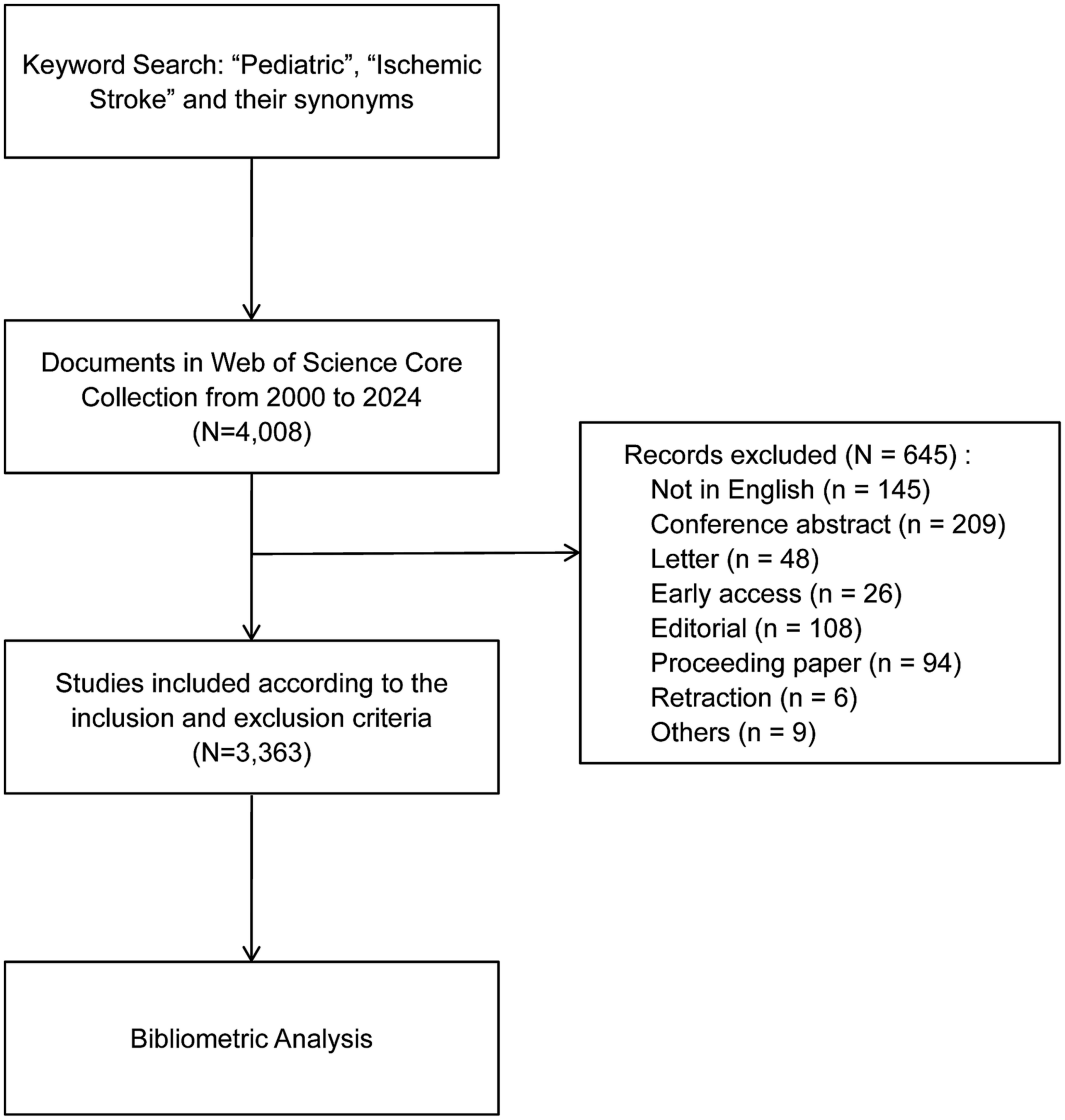
Flowchart of the study selection in pediatric ischemic stroke research.

A comprehensive literature search was conducted in the WOSCC database from January 1, 2000, to December 31, 2024. The detailed search strategy was defined as follow: TS = (“ischemic stroke” OR “cerebral ischemia” OR “ischaemic stroke” OR “brain ischemia” OR “cerebral ischaemia” OR “cerebral infarction” OR “brain ischaemia” OR “brain infarction”) AND TS = (“children” OR “child” OR “pediatric” OR “pediatrics”). Only original articles and review articles published in English were included. The selection flowchart of the included studies is presented in Figure 1. Additionally, necessary confirmations of the investigation outcomes were carried out. Additionally, a supplementary search was performed in the PubMed database using identical keywords and timeframes, with the publication type filter set to “Clinical Trial,” to identify relevant interventional studies for qualitative analysis. The processes of data extraction, cleaning, and standardization were executed by two independent researchers. Any discrepancies encountered during this process were resolved through discussion and consultation with the corresponding author. For detailed search parameters, cleaning rules, and software settings, please refer to Supplementary Table S1.

### 2.2 Bibliometric analysis based on WOSCC

Quantitative bibliometric analysis and visualization were performed using data exclusively derived from the WOSCC database, as it provides standardized citation metadata essential for network mapping. The selected records were exported in “Plain Text” format with “Full Record and Cited References”. Bibliometric data, including titles, authors, affiliations, countries, journals, and keywords, were extracted and analyzed using the R package “bibliometrix” (version 4.4.2) (11). The resulting data were then visualized using VOSviewer (version 1.6.20) to construct co-citation and collaboration networks among countries, authors, and institutions (12), and CiteSpace (version 6.2. R4) to generate a dual-map overlay of journals and to perform keyword burst analysis, thereby detecting emerging research trends and tracing thematic evolution within the field (13). In addition, the H-index was calculated to provide a composite measure of an author’s academic influence based on both publication quantity and citation impact (14).

### 2.3 Clinical analysis based on PubMed

To complement the bibliometric mapping with specific clinical insights, we conducted a qualitative content analysis of clinical trials identified through PubMed. Using identical keywords and timeframes and applying the publication type filter “Clinical Trial,” we retrieved a total of 2,556 studies related to pediatric IS, among which 59 were clinical trials. These trials were used solely for qualitative categorization and did not contribute to the bibliometric mapping. The 59 clinical trials were manually categorized according to their primary research focus. Screening and extraction were conducted independently by two researchers, with any discrepancies resolved through discussion and consensus with the corresponding author.

During the screening process, strict inclusion and exclusion criteria were applied to ensure relevance and quality of the selected studies. The inclusion criteria were as follows: (1) publications written exclusively in English; and (2) clinical trials, including randomized controlled trials and non-randomized interventional studies, were included. The exclusion criteria are as follows: (1) studies without full-text availability; (2) studies involving non-human subjects; (3) duplicates; (4) studies involving patients aged >18 years; and (5) studies on other types of strokes unrelated to ischemic stroke.

## 3 Results

### 3.1 Trends in publications and citations

**Figure 2:**
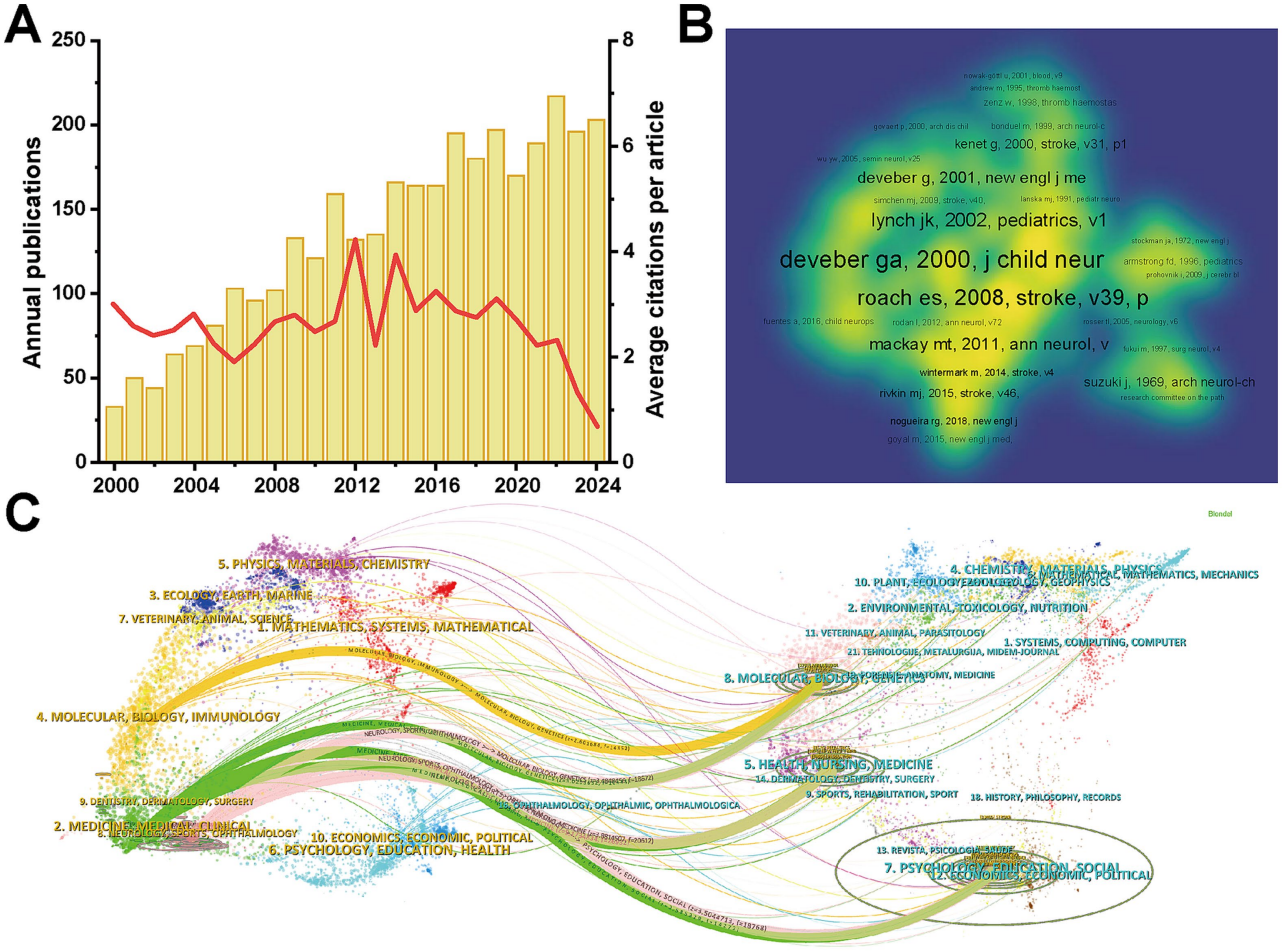
Literature and journal analysis of pediatric ischemic stroke: **(A)** Annual number of publications and average citations per year. The yellow bars represent the annual number of publications, and the red line represents the average citations per article. **(B)** Co-citation network of the publications. Labels represent highly cited references. Font size is proportional to citation frequency. The color spectrum (blue to yellow) indicates the density of co-citations, with brighter areas representing clusters of intensely co-cited research. **(C)** Dual-map overlay of journal relationships. In the visualization, yellow-labeled areas on the left represented the citing journal, while blue-labeled areas on the right corresponded to cited journals. The colored curved lines depict citation paths, illustrating the interdisciplinary flow of knowledge from source disciplines to target disciplines.

A total of 3,363 publications related to pediatric IS were retrieved from the WOSCC database, comprising 2,781 original articles and 582 review articles. Figure 2A illustrated the annual trends in publications and citations from 2000 to 2024. The number of publications exhibited a steady upward trend since 2000, with an average annual growth rate of 7.86%, highlighting the increasing academic interest in pediatric IS. The publication count peaked in 2022 (217 studies, 6.45%), followed by 2024 (203 studies, 6.04%). Collectively, all included studies accumulated 101,933 total citations, with an average of 30.31 citations per article. The years 2012 and 2014 recorded the highest average citations per publication, highlighting the lasting impact of seminal works published during that period.

### 3.2 Document analysis

Among the top 10 most cited articles, six originated from the United States, underscoring its leading role in pediatric IS research (Table 1). The most-cited article was published in *The Lancet* by Feigin et al. in 2014, with 2,861 citations, providing a comprehensive and comparative evaluation of the global disease burden of stroke (15). The second most cited paper, titled “Diagnosis and Management of Cerebral Venous Thrombosis: A Statement for Healthcare Professionals From the American Heart Association/American Stroke Association”, was authored by Saposnik et al. in 2011 and received 1,262 citations (16). In terms of research topics, half of the top ten articles were clinical statements or guidelines, with three published by the American Heart Association and two by the American College of Chest Physicians (16–20). Additionally, two studies addressed the epidemiology of stroke (15, 21). The co-citation network showed that 330 publications co-cited the pivotal study published in the *Journal of Child Neurology* in 2000 (22), underscoring its foundational influence on the pediatric IS field (Figure 2B).

**Table 1:** Top 10 most cited publications of pediatric ischemic stroke research.

Rank	Title	Institution	Authors	Journal	Citations	Year
1	Global and regional burden of stroke during 1990–2010: findings from the Global Burden of Disease Study 2010	Auckland University of Technology	Feigin VL, et al.	Lancet	2,861	2014
2	Diagnosis and Management of Cerebral Venous Thrombosis: A Statement for Healthcare Professionals From the American Heart Association/American Stroke Association	The American Heart Association	Saposnik G, et al.	Stroke	1,262	2011
3	Antithrombotic Therapy in Neonates and Children: Antithrombotic Therapy and Prevention of Thrombosis, 9th ed.: American College of Chest Physicians Evidence-Based Clinical Practice Guidelines	The American College of Chest Physicians	Monagle P, et al.	Chest	1,018	2012
4	Safety of Cell Therapy with Mesenchymal Stromal Cells (SafeCell): A Systematic Review and Meta-Analysis of Clinical Trials	University of Ottawa	Lalu MM, et al.	Plos One	855	2012
5	Cerebral Sinovenous Thrombosis in Children	Hospital for Sick Children	Deverber G, et al.	New England Journal of Medicine	773	2001
6	Management of Stroke in Infants and Children: A Scientific Statement From a Special Writing Group of the American Heart Association Stroke Council and the Council on Cardiovascular Disease in the Young	The American Heart Association	Roach ES, et al.	Stroke	699	2008
7	Age at stroke: temporal trends in stroke incidence in a large, biracial population	University of Cincinnati College of Medicine	Kissela BM, et al.	Neurology	562	2012
8	Statin Safety and Associated Adverse Events: A Scientific Statement From the American Heart Association	The American Heart Association	Newman CB, et al.	Arteriosclerosis Thrombosis and Vascular Biology	493	2019
9	Report of the National Institute of Neurological Disorders and Stroke Workshop on Perinatal and Childhood Stroke	National Institute of Neurological Disorders and Stroke	Lynch JK, et al.	Pediatrics	485	2002
10	Antithrombotic therapy in neonates and children: American College of Chest Physicians Evidence-Based Clinical Practice Guidelines (8th Edition)	The American College of Chest Physicians	Monagle P, et al.	Chest	482	2008

We also gathered 59 clinical trials from PubMed that addressed pediatric IS and arranged them into four classifications according to their research orientation (Supplementary Figure S1 and Table S2). Class 1 concentrated on the initial detection and diagnosis of pediatric IS, while Class 2 explored therapeutic approaches for pediatric IS, assessing their efficacy and prognosis. The Class 3 primary emphasis was on recovery programs and predicting outcomes subsequent to the manifestation of pediatric IS. Class 4 primarily investigated the medical characteristics of childhood IS and how particular ailments affect its occurrence, with a particular focus on sickle cell anemia. It was observed that Class 4 studies formed the majority (44.07%), followed by Class 2 studies (30.50%). Over time, the research direction of pediatric IS has gradually evolved from diagnosis to management and rehabilitation.

### 3.3 Core journal analysis

Table 2 shows the top 10 most active and influential journals in the field of pediatric IS, which collectively contributed 861 publications (25.60%). *Pediatric Neurology* published the largest number of articles (160 publications, 4.76%), followed by *Stroke* (158 publications, 4.70%) and *Journal of Child Neurology* (134 publications, 3.98%). Notably, *Stroke* received approximately twice as many citations as the second-ranked journal *Pediatrics* (9,839 vs. 4,818 citations), and had the highest H-index (H = 52) among all journals, despite ranking second in publication volume. These metrics underscored its central role as a key academic platform for disseminating high-impact research in pediatric IS. In addition, the dual-map overlay generated by Citespace illustrated the thematic distribution of citing and cited journals (Figure 2C). In the visualization, yellow-labeled areas on the left represented the citing journal, while blue-labeled areas on the right corresponded to cited journals. The main citing journals were concentrated in fields such as “Molecular, Biology, Immunology”, “Medicine, Medical and Clinical”, and “Neurology, Sports, Ophthalmology”. Correspondingly, the primary cited sources stemmed from “Molecular, Biology, Immunology”, “Health, Nursing and Medicine”, and “Psychology, Education, Social”. This pattern highlights the interdisciplinary collaborative nature of research and clinical practice in pediatric IS.

**Table 2:** Top 10 journals by number of publications in pediatric ischemic stroke.

Rank	Journal	Counts	TC	H-index	IF	JCR
1	*Pediatric Neurology*	160	3,206	31	3.2	Q1
2	*Stroke*	158	9,839	52	7.9	Q1
3	*Journal of Child Neurology*	134	2,791	30	2	Q2
4	*Childs Nervous System*	78	1,255	19	1.3	Q3
5	*Developmental Medicine and Child Neurology*	71	2,331	27	3.8	Q1
6	*Neurology*	65	4,173	28	8.4	Q1
7	*Pediatrics*	55	4,818	38	6.2	Q1
8	*Journal of Neurosurgery-Pediatrics*	48	1,084	19	2.1	Q2
9	*Journal of Stroke & Cerebrovascular Diseases*	47	432	11	2	Q3
10	*European Journal of Pediatric Neurology*	45	748	16	2.3	Q2

TC, total citations; IF, impact factor; JCR, journal citation reports.

### 3.4 Country analysis

**Figure 3:**
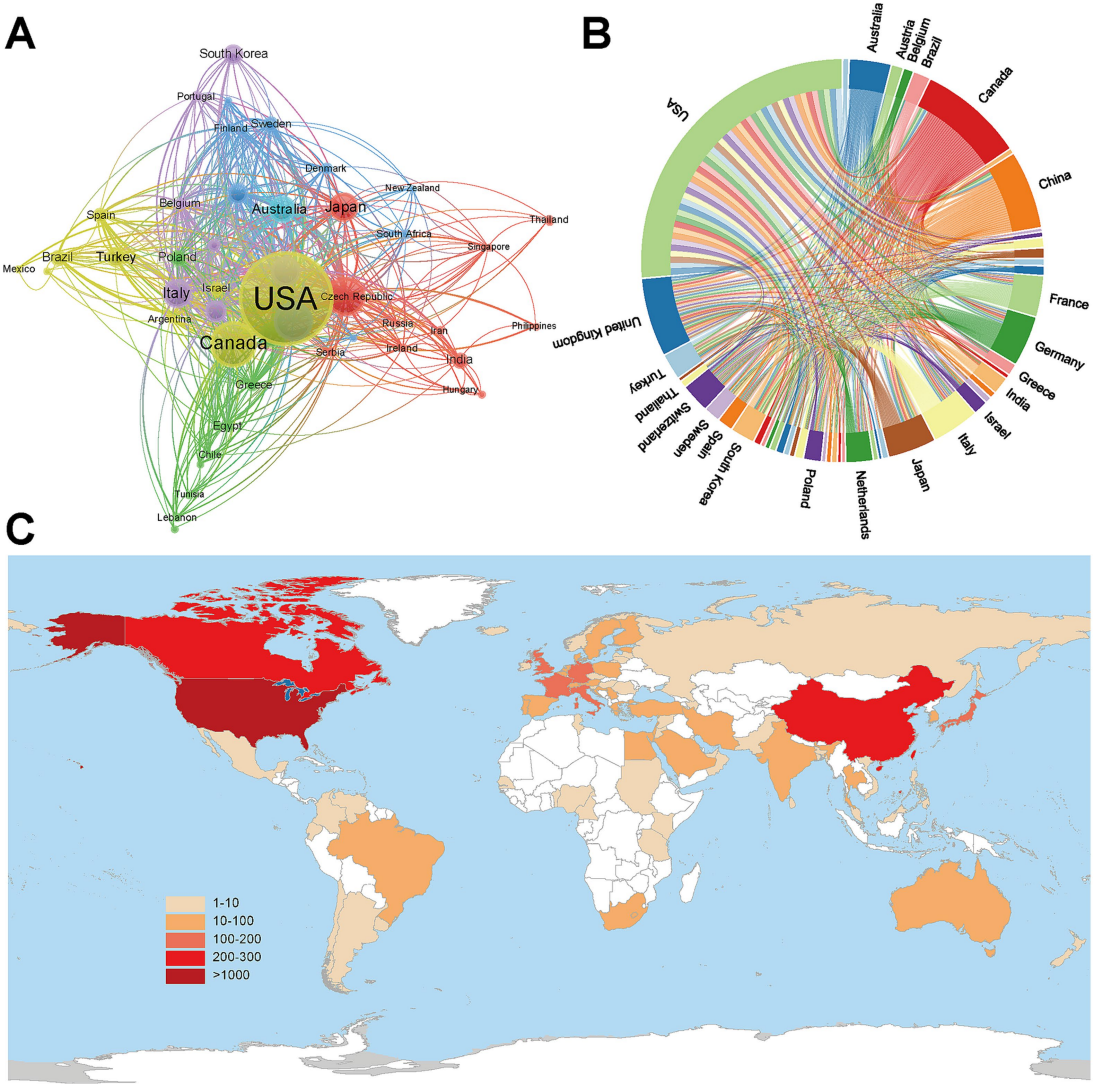
Country analysis of pediatric ischemic stroke research: **(A)** Collaboration network of different countries. Each node represents a country. The node size is proportional to the number of publications. The thickness of the connecting lines indicates the strength of academic collaboration between the two countries. Colors denote different clusters of countries that frequently collaborate. **(B)** Collaboration network among countries. The outer circular segments represent countries. The internal connecting ribbons illustrate collaborative links, with the ribbon width proportional to the volume of joint publications between countries. **(C)** Geographical distribution of major contributing countries. Color intensity (from beige to dark red) reflects the number of publications, with darker red indicating higher productivity.

A total of 94 countries contributed to pediatric IS research, and the top 10 publishing countries were listed in Table 3. The United States led the field, publishing 1,111 articles (33.04%) and accumulating 40,894 total citations. Notably, this output was more than twice the combined total of the second-ranked China (276 publications, 8.2%) and third-ranked Canada (258 publications, 7.7%), and garnered almost ten times the number of citations as China, underscoring its dominant academic influence in this research field. In terms of average citations per article, Canada ranked first (44.40 citations), followed by the United States (36.80 citations) and Australia (28.00 citations). In the global collaboration network, the United States also demonstrated the strongest total link strength, reflecting its extensive international partnerships (Figures 3A,B). Furthermore, the geographic distribution of publications revealed a concentration of research output in North America and East Asia (Figure 3C).

**Table 3:** Top 10 countries by publications output in pediatric ischemic stroke.

Rank	Country	Counts	Citations	Percentage	Citation per article
1	USA	1,111	40,894	33.0%	36.80
2	China	276	4,094	8.2%	14.80
3	Canada	258	11,467	7.7%	44.40
4	United Kingdom	168	7,419	5.0%	14.80
5	Japan	162	3,253	4.8%	20.10
6	Germany	130	3,490	3.9%	26.80
7	Italy	119	2,393	3.5%	20.10
8	France	105	2,745	3.1%	26.10
9	Australia	90	2,518	2.7%	28.00
10	Turkey	86	1,283	2.6%	14.90

**Figure 4:**
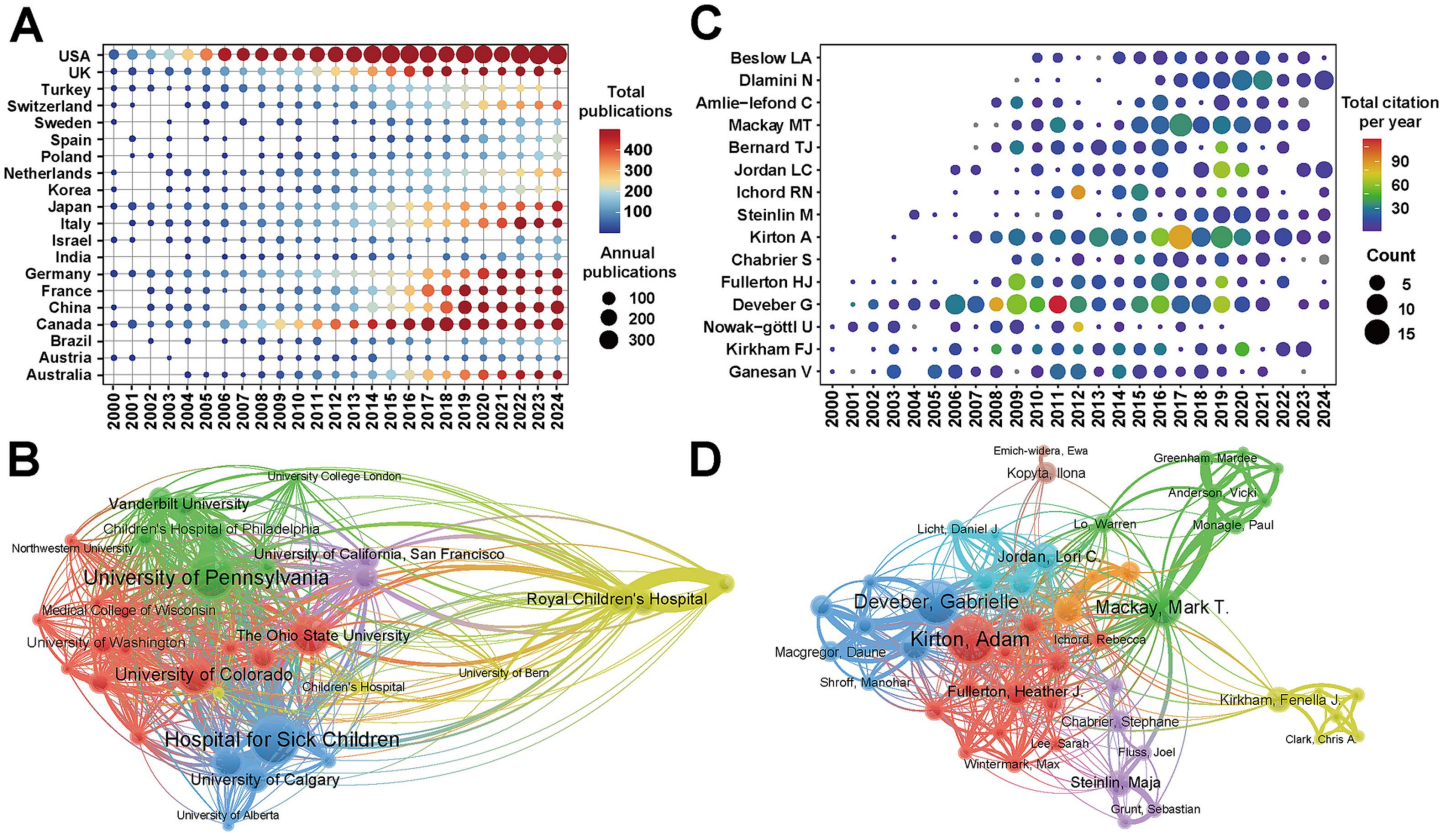
Country, institution and author analysis of pediatric ischemic stroke research: **(A)** Temporal distribution of the top 20 productive countries. The bubble size indicates the number of annual publications, while the color gradient (from blue to red) represents the total publication volume, with dark red indicating the highest overall productivity. **(B)** Institutional collaboration network. Each node represents an institution. The node size is proportional to the number of publications. The thickness of the connecting lines indicates the strength of collaborative ties between institutions. Colors represent clusters of institutions that frequently collaborate. **(C)** Publication trends of the top 20 most productive authors. The bubble size represents the author’s annual publication output. The color gradient indicates the total citations received per year, with red bubbles representing years of high citation impact. **(D)** Author collaboration network. Each node represents an author. The node size reflects the author’s publication volume. The thickness of the lines signifies the intensity of collaboration between two authors. Colors denote distinct collaborative research communities.

Figure 4A showed the annual publication trends of the top 20 countries, where the deeper red coloration of each dot indicated a higher publication count. The United States has consistently ranked first in annual output since 2000. This comparison further highlighted the exceptional contribution of the United States to this area. For China, although relevant studies appeared as early as 2000, the publication volume did not show a marked increase until 2018, indicating a growing research interest and investment in pediatric IS in recent years.

### 3.5 Institution analysis

A total of 3,574 institutions contributed to publications on pediatric IS, with the top 10 institutions ranked by publication volume presented in Table 4. Among them, three are from Canada, four are from the United States and two are from Australia. The Hospital for Sick Children ranked first with 172 publications (5.11%), followed by the University of Toronto (123 publications, 3.66%), and the University of Pennsylvania (120 publications, 3.57%). In terms of total citations, the Hospital for Sick Children again led the field with 9,947 citations, followed by the University of California, San Francisco (9,422 citations), and the University of Toronto (7,301 citations). Notably, both the Hospital for Sick Children and the University of Pennsylvania exhibited the strongest total link strength, highlighting the pivotal leadership roles in research collaboration (Figure 4B). These results indicate that Canadian institutions, particularly the Hospital for Sick Children, are not only major contributors but also central nodes in the collaboration network of pediatric IS. Despite the strong domestic networks observed, international collaboration remains limited, which may hinder the global advancement of research in pediatric IS.

**Table 4:** Top 10 institutions by publications output in pediatric ischemic stroke.

Rank	Institution	Country	Counts	Total citations
1	Hospital for Sick Children	Canada	172	9,947
2	University of Toronto	Canada	123	7,301
3	University of Pennsylvania	USA	120	5,787
4	University of California San Francisco	USA	117	9,422
5	University of Calgary	Canada	107	5,399
6	Johns Hopkins University	USA	93	3,400
7	Royal Children’s Hospital	Australia	83	3,214
8	University College London	UK	82	4,185
9	University of Colorado	USA	81	3,520
10	University of Melbourne	Australia	80	4,768

### 3.6 Author contributions

A total of 15,227 authors participated in the study of pediatric IS, with the top 20 most prolific authors illustrated in Figure 4C. During the first twelve years (2000–2011), Gabrielle deVeber published the most articles (50 publications, 1.49%), followed by Vijeya Ganesan (29 publications, 0.86%). Notably, a 2011 guideline co-authored by Gabrielle deVeber received the highest average citations per year (118.6 citations), reflecting its enduring influence (16). Since 2012, the number of publications has continued to rise, with Adam Kirton emerging as the most productive authors (85 publications, 2.53%), followed by Nomazulu Dlamini (50 publications, 1.49%). The top 15 authors ranked by H-indices were shown in Table 5, with nearly half based in the United States (7/15). Gabrielle deVeber, affiliated with the University of Toronto, held the highest H-index (H-index = 49), followed by Adam Kirton (H-index = 41) and Heather J Fullerton (H-index = 34). In the author collaboration network, Gabrielle deVeber and Adam Kirton, both from Canada, exhibited the strongest total link strength, underscoring their prominent leadership roles in advancing research and fostering collaboration in pediatric IS (Figure 4D).

**Table 5:** Top 15 authors ranked by H-index in pediatric ischemic stroke research.

Authors	Country	H-index	Counts	Percentage	Total citations
Gabrielle A DeVeber	Canada	49	138	4.10%	12,593
Adam Kirton	Canada	41	106	3.15%	5,032
Heather J Fullerton	USA	34	54	1.61%	4,739
Fenella Jane Kirkham	UK	33	53	1.58%	5,000
Vijeya Ganesan	UK	27	59	1.75%	3,266
Mark T Mackay	Australia	27	72	2.14%	2,310
Timothy J Bernard	USA	25	46	1.37%	2,350
Michael Dowling	USA	25	33	0.98%	1,656
Rebecca N. Ichord	USA	23	38	1.13%	3,131
Lori C Jordan	USA	23	52	1.55%	2,181
U Nowak-Göttl	Germany	22	34	1.01%	3,025
Maja Steinlin	Switzerland	22	46	1.37%	1,629
Catherine Amlie-Lefond	USA	20	35	1.04%	1,684
Nomazulu Dlamini	Canada	19	54	1.61%	1,032
Neil A Goldenberg	USA	19	28	0.83%	2,334

### 3.7 Keywords and research trends

**Figure 5:**
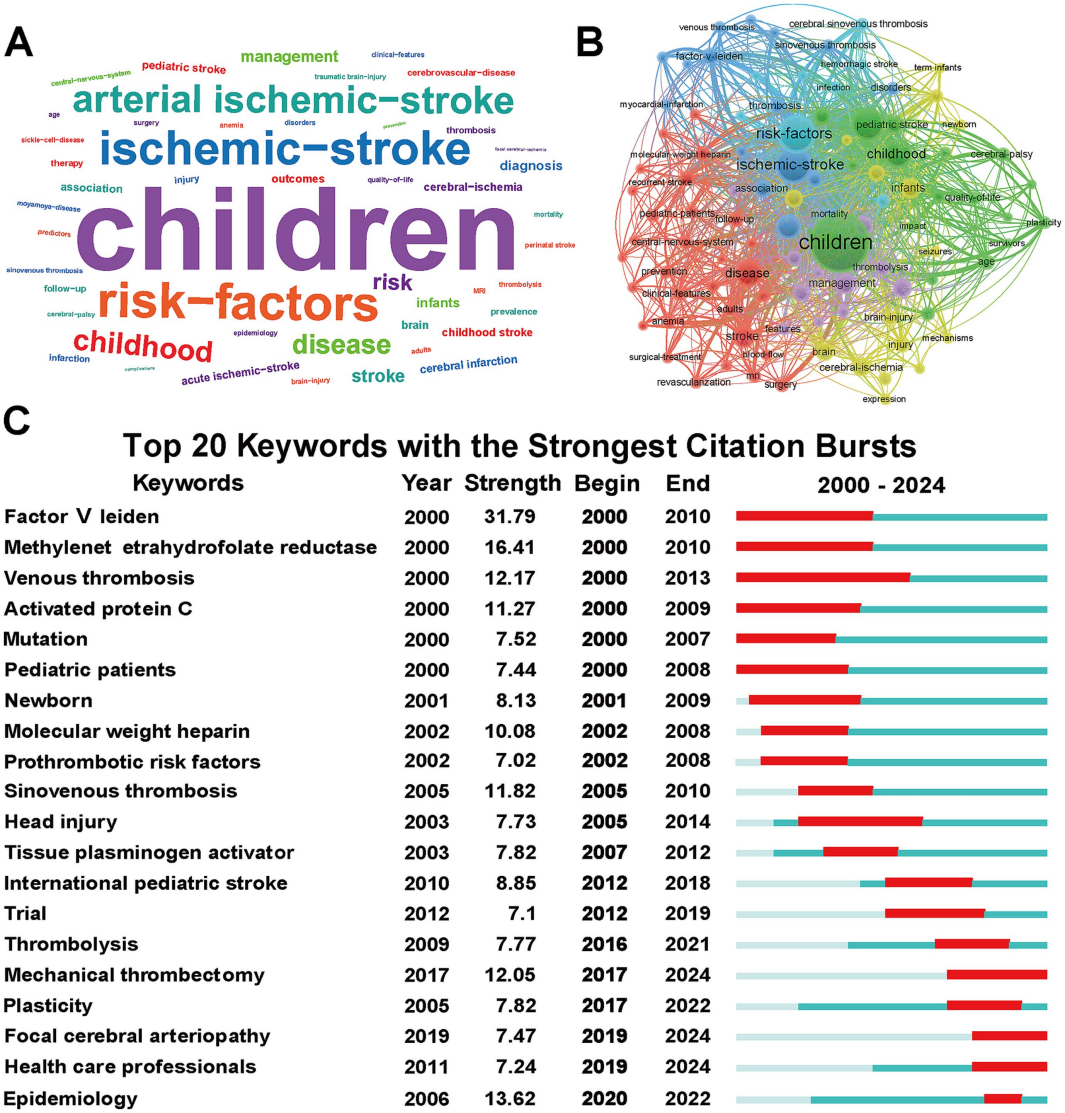
Keyword analysis of pediatric ischemic stroke research: **(A)** Keywords plus word cloud ranked by frequency. The font size of each term is directly proportional to its frequency of occurrence. **(B)** Co-occurrence network of keywords plus terms. Each node represents a keyword. The node size reflects the frequency of the keyword mechanical thrombolysis. The thickness of the connecting lines indicates the strength of the co-occurrence relationship (how often two terms appear together in the same publication). Colors denote distinct clusters of closely related research topics. **(C)** Top 20 keywords with the strongest citation bursts. The red bars indicate the duration of the citation burst, marking periods where a specific keyword received a sudden surge in research attention. The blue line represents the timeline from 2000 to 2024. Keywords are sorted by the year the burst started.

Figure 5A presented the top 50 most frequently occurring terms in WOSCC database, with “Children” being the only keyword that appeared more than one thousand times (1,372 times). Other high-frequency terms included “Risk-factors,” “Ischemic-stroke,” “Arterial ischemic-stroke,” “Childhood,” “Disease,” and “Management.” Concurrently, we confirmed these findings by extracting keywords from all pertinent studies on PubMed and generating a word cloud visualization (Supplementary Figure S2). Current research on pediatric IS is primarily concerned with its clinical diagnosis and management.

In the keyword co-occurrence analysis, all keywords were classified into five thematic clusters, each represented by a different color to delineate major research domains (Figure 5B). The largest red clusters included terms such as “Stroke,” “Disease,” “Clinical-features,” “Cerebral-features,” reflecting a focus on the clinical presentation of pediatric IS. The green cluster comprised “Children,” “Childhood,” “Pediatric stroke,” “Quality-of-life,” and “Impact,” indicating a research focus on the long-term outcomes and quality of life implications of pediatric IS. The blue cluster centered on “Risk-factors,” “Disorders,” “Infection,” “Thrombosis,” and “Factor V Leiden,” indicating a strong interest in the etiology and risk factors associated with pediatric IS.

Furthermore, keyword burst analysis was conducted to identify emerging research hotspots of pediatric IS (Figure 5C). Among the top 20 keywords with the strongest citation bursts, “Factor V Leiden” exhibited the highest burst strength (31.79), followed by “Methylenet etrahydrofolate reductase” (16.41) and “Epidemiology” (13.62). Notably, keywords such as “Mechanical thrombectomy,” “Focal cerebral arteriopathy,” and “Health care professionals” continued to show strong bursts in 2024, underscoring current priorities in exploring etiological mechanisms, advancing clinical interventions, and promoting multidisciplinary collaboration in pediatric IS research.

## 4 Discussion

### 4.1 General information

Reflecting the growing global interest in pediatric IS, at the international level, the United States, China, and Canada have emerged as major contributors to pediatric IS research. Among them, the United States stands out with the highest research outputs and the most extensive collaborative networks, underscoring its leading influence in this domain. Although the United Kingdom demonstrates a moderate publication volume, its strong collaboration with multiple countries contributes to synergistic international efforts in addressing shared research challenges. In terms of institutional contributions, the Hospital for Sick Children in Canada leads with the highest number of publications and citations, highlighting its prominent academic impact. Despite China ranking second in total publication volume, no Chinese institutions appear among the top ten most productive countries, suggesting that pediatric IS research in China remains fragmented and lacks centralized leadership from core institutions. This suggests a relative fragmentation in China, indicating the need to establish dedicated centers to consolidate expertise and resources, thereby promoting more focused and collaborative investigations in this field. Additionally, enhancing international collaborations is crucial for integrating diverse perspectives and accelerating scientific research progress in pediatric IS.

From the perspective of authorship, Canadian scholars, particularly Gabrielle deVeber and Adam Kirton, have made substantial contributions through high-impact publications to the development of this area. Notably, a seminal study by Gabrielle deVeber in 2000 is co-cited by 9.81% of the included literature (22), offering a comprehensive assessment of neurological outcomes and associated risk factors in children with IS. This study continues to serve as a foundational reference for clinical research and practice. Meanwhile, the significant rise in publication output by Chinese scholars since 2018 reflects both growing interest and rapid development in this area within China.

Regarding academic journals, *Pediatric Neurology* leads in the number of publications, whereas *Stroke* ranks highest in total citations and H-index, indicating their roles as core platforms for disseminating influential research in pediatric IS. The top ten journals in this field primarily focus on neuroscience and children’s brain disorders, which is essential for advancing understanding of the pathophysiological mechanisms of pediatric IS. Furthermore, research on pediatric IS is highly interdisciplinary, intersecting with neuroscience, rehabilitation medicine, cardiovascular medicine, and nursing. Given its association with severe long-term sequelae, multidisciplinary involvement including clinicians, rehabilitation therapists, and nursing professionals is frequently emphasized in the literature, reflecting recognition of its importance for advancing research and supporting patient care in this field.

### 4.2 Hotspots and frontiers

Based on the keyword analysis, current research hotspots in pediatric IS primarily center around three areas: etiological and pathological mechanisms, the advancement of endovascular treatment strategies, and the development of multidisciplinary healthcare teams.

Focal cerebral arteriopathy (FCA), a major contributor to and recurrence predictor of pediatric arterial ischemic stroke (PAIS) (23), is detected in approximately 64% of previously healthy children presenting with IS (4). The diagnosis of FCA is based on vascular imaging findings, which are pathologically characterized by unilateral stenosis or irregularity of the cerebral arteries (24). Pathologically, FCA is associated with an inflammatory response associated with infection, which in turn contributes to PAIS (23, 25, 26). Factor V Leiden (FV_Leiden_), a common hereditary thrombophilia, results from a mutation in the factor V gene (27). This mutation then confers resistance to inactivation by activated protein C (APC), thereby creating a hypercoagulable state that significantly increases the risk of thromboembolic events (27). A previous study reported a markedly higher prevalence of FV_Leiden_ mutation among children with IS compared to children without IS (23.3%, *p* < 0.05), indicating its potential role as a genetic risk factor of pediatric stroke (28). In our study, FV_Leiden_ emerged as the keyword with the strongest citation burst, highlighting growing attention to the hereditary prothrombin pathway in the pathogenesis of pediatric IS. Together, both FV_Leiden_ and FCA represent critical frontiers in pediatric IS research, underscoring a sustained focus on identifying and characterizing risk factors of pediatric IS.

In adult IS, intravenous thrombolysis (IVT) administered within 4.5 h of symptom onset and endovascular thrombectomy (EVT) within 24 h are the two standardized treatments recommended by current clinical guidelines (29). However, in pediatric patients, the immaturity of the fibrinolytic system renders the optimal dosage of recombinant tissue plasminogen activator (rt-PA) uncertain, and related clinical trials have been discontinued due to insufficient enrollment and limited safety data (30, 31).

In 2015, several landmark randomized controlled trials published in the *New England Journal of Medicine* demonstrated that EVT offers superior efficacy and outcomes compared to IVT in adults (32–34). Due to the lack of pediatric-specific treatment guidelines, clinical practice in pediatric IS has often relied on extrapolating from adult protocols (35). Consequently, the American Heart Association updated its guidelines to acknowledge the potential applicability of adult EVT protocol in patients under 18 years of age (36). Since then, mechanical thrombectomy has been increasingly reported in pediatric patients (37).

Previous clinical studies in children with large-vessel occlusions (e.g., basilar and middle cerebral arteries) have reported that the application of mechanical thrombectomy is associated with higher recanalization rates, reduced procedural risk, and improved cost-effectiveness (38, 39). Importantly, data from Save ChildS Study indicate that, although the median age of treated children was 11.3 years, endovascular recanalization has been performed in selected cases as young as 0.8 years (8 months), with reported lower age thresholds extending up to 6 years (40). Therefore, the youngest treated age at which mechanical thrombectomy may be performed appears to vary and may depend on institutional expertise, technical feasibility, and peri-procedural support rather than fixed age-based criteria. While the growing publication volume reflects sustained academic interest in investigating its efficacy and safety in pediatric IS, discussions regarding complications and evidence gaps remain ongoing (41). Consistent with this trend, our bibliometric analysis identifies mechanical thrombectomy as a prominent research focus in recent years, indicating a growing clinical interest in its applicability within the pediatric IS field. Nevertheless, further multicenter prospective studies are required to strengthen the evidence base, refine patient selection criteria, and clarify the role of mechanical thrombectomy in pediatric IS.

Although the overall prognosis of pediatric IS is more favorable than that in adults, the recurrence rate remains high, reaching up to 25%, and over 20% of affected children suffer from severe neurological deficits (42). Consequently, the economic burden on families of pediatric patients is often greater than that associated with adult stroke cases (43). To address this, early mobilization of specialized healthcare resources and the establishment of multidisciplinary collaborative models have been emphasized in the literature for the timely detection and treatment of pediatric IS (44). The emergence of the keyword “health care professionals” as a recent hotspot highlights a shifting research trend towards multidisciplinary collaborative models in managing pediatric IS. For example, the American Stroke Association, founded in 1998, is the largest and most influential organization dedicated to the prevention and treatment of stroke.^1^ It provides scientific, systematic, and standardized diagnostic and therapeutic protocols, which are regularly updated and have significantly advanced global efforts in stroke prevention, treatment, and rehabilitation (45). Recently, Boston Children’s Hospital has formed an acute stroke response team comprising cerebrovascular specialists, neuroradiologists, intensivists, and anesthesiologists, enabling rapid assessment and intervention within critical therapeutic windows (46). However, dedicated multidisciplinary stroke management teams focused on pediatric patients remain scarce (44). Greater emphasis has been noted in the literature regarding the development of pediatric stroke centers, reflecting recognition of their potential role in standardizing care for children affected by IS (47).

### 4.3 Advantages and limitations

Compared with earlier citation-based summaries, our study extends beyond prior citation-based overviews, such as the study by Panagopoulos et al., which summarized the 50 most cited articles in pediatric arterial ischemic stroke but did not conduct a comprehensive bibliometric or network-based analysis (48). Specifically, our study captures the most recent two decades of research from 2000 to 2024 and provides a comprehensive, field-level synthesis of the landscape. Unlike a traditional review, this high-level “map” serves as a foundational roadmap for newcomers entering the field and a strategic guide for established groups seeking collaborative opportunities. To ensure the robustness and validity of these findings, we employed a multi-tool triangulation approach utilizing Bibliometrix, VOSviewer, and CiteSpace, complemented by a dual-database search strategy integrating WOSCC and PubMed. Furthermore, our analysis offers actionable signals regarding core journals and institutional networks, providing researchers with data-driven guidance for manuscript submission strategies and identifying global centers of excellence for future multi-center trials.

However, this study also has several limitations. First, the literature was retrieved exclusively from the WOSCC and PubMed, excluding other databases such as Scopus and Embase, which could lead to incomplete coverage. Scopus includes a more expanded journal coverage and citation analysis capabilities than PubMed and Web of Science, particularly for non-English and regional literature (49). Embase, while widely recognized as a robust supplement to PubMed in health sciences, has stronger coverage for pharmacological and biomedical indexing but relies on a single discipline, which may compromise the representativeness of the results (50, 51). Consequently, certain non-Western countries, regional journals, or discipline-specific publications may be underrepresented in our dataset. With respect to the suggested limited robustness check, we carefully considered comparing rankings derived from WOSCC-only data with those from a combined database set. However, as all quantitative bibliometric mapping, network construction, and citation-based analyses in the present study were intentionally restricted to WOSCC to ensure metadata consistency and methodological comparability, incorporating additional databases such as Scopus or Embase would require re-running the full retrieval, de-duplication, and network reconstruction pipeline. Moreover, prior comparative studies have shown that Scopus and the Web of Science exhibit highly similar patterns in geographical and disciplinary coverage, particularly with respect to the over-representation of Europe, North America, and other high-income regions (52). Taken together, while excluding Scopus and Embase may reduce overall coverage completeness, the relative rankings of leading countries and core journals derived from WOSCC-based analyses are unlikely to be materially affected. Second, recently published articles tend to have fewer citations, which may affect the accuracy and timeliness of citation-based indicators. Third, the bibliometric analysis relied on software-based algorithms, which may introduce certain biases in clustering and visualization.

Future studies should aim to integrate data from multiple databases to ensure more comprehensive coverage. Additionally, larger-scale, multicenter, and prospective studies are needed to validate current findings, thereby supporting the development of refined, evidence-based clinical guidelines for the diagnosis, treatment, and management of pediatric IS.

## 5 Conclusion

This study provides a bibliometric analysis to systematically explore research trends and development in pediatric IS. Over the past two decades, the number of publications in this field exhibited a continuous upward trajectory, highlighting increasing global attention. Current research hotspots primarily cluster around the pathological mechanisms and evolving management strategies of pediatric IS. Additionally, enhanced international and institutional collaboration is frequently emphasized in the literature to facilitate the global exchange of scientific knowledge and optimize the allocation of research resources.

## Data Availability

The original contributions presented in the study are included in the article/Supplementary material, further inquiries can be directed to the corresponding authors.

## Footnotes

1
https://www.stroke.org/en/

## Supplementary material

SUPPLEMENTARY FIGURE S1 Clinical trial of pediatric ischemic stroke in PubMed from 2000 to 2024. Colored dots distinguish the trials by specific classification (Class 1–4), illustrating the evolving landscape of clinical research over the past two decades.

SUPPLEMENTARY FIGURE S2 Word cloud of the keywords related to pediatric ischemic stroke on PubMed ranked by frequency. The font size of each term is directly proportional to its frequency of occurrence.
