# Supplementary figures and images for: Global insights into pediatric ischemic stroke: a bibliometric and visualization analysis
Source: Front Med (Lausanne). 2026 Mar 16;13:1708673. doi: 10.3389/fmed.2026.1708673 (PMC13033810; doi:10.3389/fmed.2026.1708673)

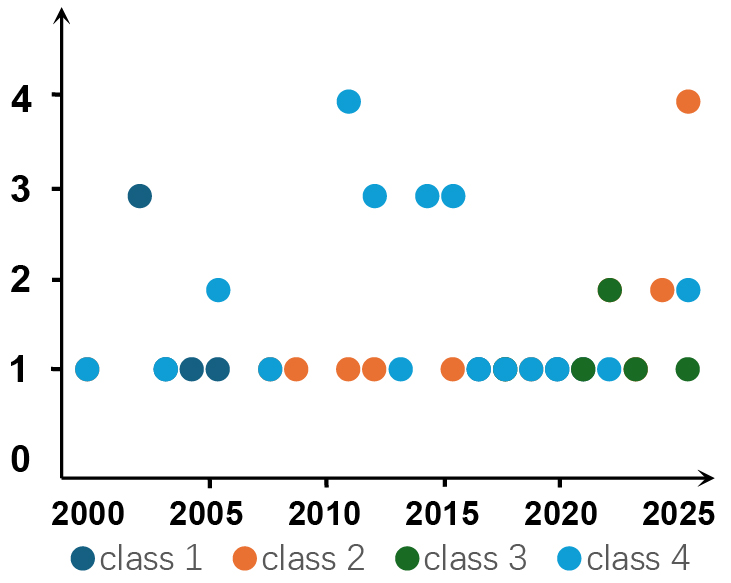

Supplement: SUPPLEMENTARY FIGURE S1 — Clinical trial of pediatric ischemic stroke in PubMed from 2000 to 2024. Colored dots distinguish the trials by specific classification (Class 1–4), illustrating the evolving landscape of clinical research over the past two decades. [file Image_1.jpeg]

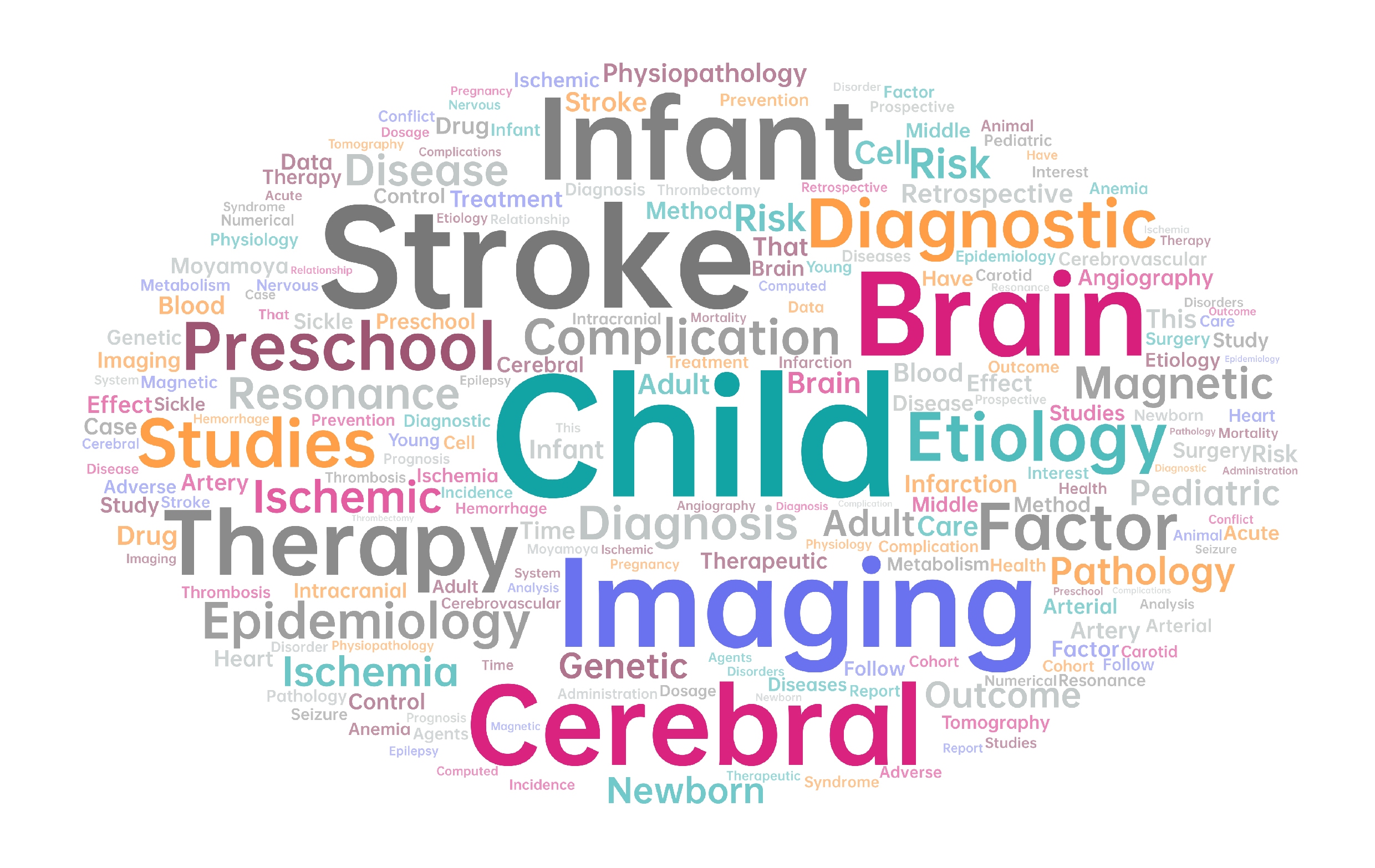

Supplement: SUPPLEMENTARY FIGURE S2 — Word cloud of the keywords related to pediatric ischemic stroke on PubMed ranked by frequency. The font size of each term is directly proportional to its frequency of occurrence. [file Image_2.jpeg]
