# Supplementary material for: Global insights into pediatric ischemic stroke: a bibliometric and visualization analysis
Source: Front Med (Lausanne). 2026 Mar 16;13:1708673. doi: 10.3389/fmed.2026.1708673 (PMC13033810; doi:10.3389/fmed.2026.1708673)
Supplement: Supplementary file 3 [file Table_1.docx]

**Supplementary File S1. Detailed Protocol for Data Retrieval, Pre-processing, and Bibliometric Analysis**

**1. Data Sources and Retrieval Strategy**

**1.1. Web of Science Core Collection (WoSCC)**

**① Retrieval Date:** April 25, 2025

**② Indexes Included:** Science Citation Index Expanded (SCI-EXPANDED)、Social Sciences Citation Index (SSCI)

**③ Search Query:** The field tag TS (TS = Topic) searches Title, Abstract, Author Keywords, and Keywords Plus.

**④ Query String:** TS = (“ischemic stroke” OR “cerebral ischemia” OR “ischaemic stroke” OR “brain ischemia” OR “cerebral ischaemia” OR “cerebral infarction” OR “brain ischaemia” OR “brain infarction”) AND TS = (“children” OR “child” OR “pediatric” OR “pediatrics”)

**⑤ Filters and Restrictions:**

**Document Types:** Article or review article.

**Language:** English.

**Time Span:** January 1, 2000 – December 31, 2024.

**1.2. PubMed Database**

**① Retrieval Date:** April 25, 2025

**② Search Strategy:** Title/Abstract search using identical terms to identify clinical trials (Table S1).

**Table S1. Search strategy in PubMed**

| **Steps** | **Strategies** |
| --- | --- |
| #1 | (ischemic stroke[Title/Abstract]) OR (cerebral ischemia[Title/Abstract]) OR (ischaemic stroke[Title/Abstract]) OR (brain ischemia[Title/Abstract]) OR (cerebral ischaemia[Title/Abstract]) OR (cerebral infarction[Title/Abstract]) OR (brain ischaemia[Title/Abstract]) OR (brain infarction[Title/Abstract]) |
| #2 | (children[Title/Abstract]) OR (child[Title/Abstract]) OR (pediatric[Title/Abstract]) OR (pediatrics[Title/Abstract]) |
| #3 | #1 OR #2 OR #3 |

**③ Filters and Restrictions:**

**Document Types:** Clinical trials.

**Language:** English.

**Time Span:** January 1, 2000 – December 31, 2024.

**2. Data Pre-processing and Cleaning Rules**

Raw bibliographic data were exported from the WoSCC in "Plain Text" format (WoSCC) with “Full Record and Cited References”, and data from PubMed were exported to EndNote for screening and manual sub-classification.

**2.1. Synonym Merging**

**① Geographical Standardization**

A. “North Ireland,” “Wales,” “England,” “Scotland,” and “UK” were merged into “United Kingdom.”

B. “People R China,” “Taiwan,” “Hong Kong,” and “Macao” were merged into “China.”

**② Keyword Standardization**

A. “Ischaemic stroke” was merged into “Ischemic stroke.”

B. “Paediatric”, “Pediatric” and “Child” were merged into “Children.”

C. “Risk factor” and “Risk factors” were merged into “Risk-factors.”

**③ Institution Standardization**

A. “Hosp Sick Children,” “SickKids,” and “Hosp Sick Children Toronto” were merged into “Hospital for Sick Children”.

B. “Univ Toronto” was merged into “University of Toronto”.

C. “Univ Penn” was merged into “University of Pennsylvania”.

**④ Author Name Standardization and Disambiguation:**

A. Authors with inconsistent middle initials or spelling formats were merged. For example, “deveber, gabrielle” and “deveber, gabrielle a.” were merged into “DeVeber, Gabrielle” to ensure accurate citation counting.

B. Authors with identical initials (e.g., “Wang Y”) were manually checked against affiliation data to separate distinct individuals.

**3. Software Parameter Settings**

**3.1. VOSviewer (Version 1.6.20)**

**Counting Method for all the analyses: Full counting**

**① References Co-citation Network**

Minimum number of citations of a cited reference: 20

Number of cited references selected: All meeting threshold

**② Keywords Co-occurrence Network**

Unit of analysis: Keywords Plus

Minimum number of occurrences of a keyword: 5

Number of keywords selected: 100

**③ Countries Collaboration Network:**

Minimum number of documents of a country: 3

Minimum number of citations of a country: 0

Number of countries selected: 50

**④ Institutions Collaboration Network:**

Minimum number of documents of an organization: 15

Minimum number of citations of an organization: 0

Number of organizations selected: 60

**⑤ Authors Collaboration Network:**

Minimum number of documents of an author: 5

Minimum number of citations of an author: 0

Number of authors selected: 50

**3.2. CiteSpace (Version 6.2.R4)**

**A. General Settings**

**Time Slicing:** From 2000 Jan to 2024 Dec (Years Per Slice: 1).

**Node Selection Criteria:** Selection Strategy: g-index; Scale factor: k = 25

**Pruning:** Pruning sliced networks

**B. Settings for Dual-Map Overlay**

**Analytic Module:** "JCR Journal Maps" in the "Overlay Maps" function.

**Method:** The overlay was generated using the z-score standardization method to visualize citation trajectories from citing journals (left) to cited journals (right).

**C. Settings for Keywords Citation Bursts Map**

**Detection Algorithm:** Kleinberg’s algorithm

**Node Type:** Keywords.

**Burstness Parameters:** Gamma=0.1; Minimum Duration: 2; Keywords included: 20
