# Supplementary material for: Global insights into pediatric ischemic stroke: a bibliometric and visualization analysis
Source: Front Med (Lausanne). 2026 Mar 16;13:1708673. doi: 10.3389/fmed.2026.1708673 (PMC13033810; doi:10.3389/fmed.2026.1708673)
Supplement: Supplementary file 4 [file Table_2.docx]

**Supplementary Table S2. Clinical trials classifications in PubMed**

| **Title** | **Year** | **Author** | **Classification** |
| --- | --- | --- | --- |
| Silent infarcts in children with sickle cell anemia and abnormal cerebral artery velocity | 2001 | Pegelow, C. H.,et al | 4 |
| Aspirin versus low-dose low-molecular-weight heparin: antithrombotic therapy in pediatric ischemic stroke patients: a prospective follow-up study | 2001 | Sträter, R.,et al | 2 |
| Cranial ultrasonography has a low sensitivity for detecting arterial ischemic stroke in term neonates | 2003 | Golomb, M. R.,et al | 1 |
| General movements detect early signs of hemiplegia in term infants with neonatal cerebral infarction | 2003 | Guzzetta, A.,et al | 1 |
| EEG and ischemic stroke in full-term newborns | 2003 | Selton, D.,et al | 1 |
| Responses of children with cerebral palsy to arm-crank exercise in the heat | 2004 | Maltais, D.,et al | 4 |
| Assessment of cerebral hemodynamics in childhood moyamoya disease using a quantitative and a semiquantitative IMP-SPECT study | 2004 | Saito, N.,et al | 1 |
| Can peak systolic velocities be used for prediction of stroke in sickle cell anemia? | 2005 | Jones, A.,et al | 1 |
| Metabolic and genetic risk factors for migraine in children | 2006 | Bottini, F.,et al | 4 |
| Symptomatic epilepsy in children with poroencephalic cysts secondary to perinatal middle cerebral artery occlusion | 2006 | Guzzetta, F.,et al | 4 |
| Homocysteine, MTHFR C677 T, vitamin B12, and folate levels in Thai children with ischemic stroke: a case-control study | 2006 | Sirachainan, N.,et al | 1 |
| Effect of preoperative skull block on pediatric moyamoya disease | 2008 | Ahn, H. J.,et al | 4 |
| Contralesional repetitive transcranial magnetic stimulation for chronic hemiparesis in subcortical paediatric stroke: a randomised trial | 2008 | Kirton, A.,et al | 2 |
| Thrombolysis in acute childhood stroke: design and challenges of the thrombolysis in pediatric stroke clinical trial | 2009 | Amlie-Lefond, C.,et al | 2 |
| Antithrombotic treatments, outcomes, and prognostic factors in acute childhood-onset arterial ischaemic stroke: a multicentre, observational, cohort study | 2009 | Goldenberg, N. A.,et al | 2 |
| Discontinuing prophylactic transfusions increases the risk of silent brain infarction in children with sickle cell disease: data from STOP II | 2011 | Abboud, M. R.,et al | 4 |
| Silent cerebral infarcts occur despite regular blood transfusion therapy after first strokes in children with sickle cell disease | 2011 | Hulbert, M. L.,et al | 2 |
| [Clinical-encephalographic evaluation of preterm children treated with cytoflavin during the first year of life] | 2011 | Rogatkina, S. O.,et al | 4 |
| Plasma glial fibrillary acidic protein levels in children with sickle cell disease | 2011 | Savage, W. J.,et al | 4 |
| Cervical carotid artery disease in sickle cell anemia: clinical and radiological features | 2011 | Telfer, P. T.,et al | 4 |
| Associated risk factors for silent cerebral infarcts in sickle cell anemia: low baseline hemoglobin, sex, and relative high systolic blood pressure | 2012 | DeBaun, M. R.,et al | 4 |
| The pediatric stroke outcome measure: a validation and reliability study | 2012 | Kitchen, L.,et al | 2 |
| Cerebral tissue hemoglobin saturation in children with sickle cell disease | 2012 | Quinn, C. T.,et al | 4 |
| Stroke in children with posterior fossa brain malformations, hemangiomas, arterial anomalies, coarctation of the aorta and cardiac defects, and eye abnormalities (PHACE) syndrome: a systematic review of the literature | 2012 | Siegel, D. H.,et al | 4 |
| Cerebral regional oxygen saturation monitoring in pediatric malfunctioning shunt patients | 2013 | Abramo, T. J.,et al | 4 |
| Neurological injury after neonatal cardiac surgery: a randomized, controlled trial of 2 perfusion techniques | 2014 | Algra, S. O.,et al | 4 |
| Headache and migraine in children with sickle cell disease are associated with lower hemoglobin and higher pain event rates but not silent cerebral infarction | 2014 | Dowling, M. M.,et al | 4 |
| Silent cerebral infarction, income, and grade retention among students with sickle cell anemia | 2014 | King, A. A.,et al | 4 |
| Association of alpha-thalassemia, TNF-alpha (-308G>A) and VCAM-1 (c.1238G>C) gene polymorphisms with cerebrovascular disease in a newborn cohort of 411 children with sickle cell anemia | 2015 | Belisário, A. R.,et al | 4 |
| Silent cerebral infarcts in very young children with sickle cell anaemia are associated with a higher risk of stroke | 2015 | Cancio, M. I.,et al | 4 |
| Safety of primed repetitive transcranial magnetic stimulation and modified constraint-induced movement therapy in a randomized controlled trial in pediatric hemiparesis | 2015 | Gillick, B. T.,et al | 2 |
| Efficacy of cytoflavin in the treatment of hypoxic-ischemic lesions of the nervous system in 1-month old children with transient neonatal hypothyroidism | 2015 | Strukov, V. I.,et al | 4 |
| Transcranial doppler re-screening of subjects who participated in STOP and STOP II | 2016 | Adams, R. J.,et al | 1 |
| Multimodal Outcome at 7 Years of Age after Neonatal Arterial Ischemic Stroke | 2016 | Chabrier, S.,et al | 4 |
| Changes in the living arrangement and risk of stroke in Japan; does it matter who lives in the household? Who among the family matters? | 2017 | Eshak, E. S.,et al | 4 |
| Early Intensive Leg Training to Enhance Walking in Children With Perinatal Stroke: Protocol for a Randomized Controlled Trial | 2017 | Hurd, C.,et al | 3 |
| Treatment-Related Noncontiguous Radiologic Changes in Children With Diffuse Intrinsic Pontine Glioma Treated With Expanded Irradiation Fields and Antiangiogenic Therapy | 2017 | Patay, Z.,et al | 2 |
| Children with sickle cell anemia with normal transcranial Doppler ultrasounds and without silent infarcts have a low incidence of new strokes | 2018 | Jordan, L. C.,et al | 4 |
| A randomised double blind placebo controlled phase 2 trial of adjunctive aspirin for tuberculous meningitis in HIV-uninfected adults | 2018 | Mai, N. T. H.,et al | 2 |
| The relationship between the prognosis of children with acute arterial stroke and polymorphisms of CDKN2B, HDAC9, NINJ2, NAA25 genes | 2019 | Bozpolat, A.,et al | 1 |
| Genetic modulators of fetal hemoglobin expression and ischemic stroke occurrence in African descendant children with sickle cell anemia | 2019 | Nicolau, M.,et al | 4 |
| MRI Vessel Wall Enhancement and Other Imaging Biomarkers in Pediatric Focal Cerebral Arteriopathy-Inflammatory Subtype | 2020 | Perez, F. A.,et al | 1 |
| Health-Related Quality of Life in Young Adults Following Pediatric Arterial Ischemic Stroke | 2020 | Rohner, A.,et al | 3 |
| Thromboprophylaxis for Children Post-Fontan Procedure: Insights From the UNIVERSE Study | 2021 | McCrindle, B. W.,et al | 4 |
| Social Cognitive Dysfunction Following Pediatric Arterial Ischemic Stroke: Evidence From a Prospective Cohort Study | 2021 | Ryan, N. P.,et al | 3 |
| Efficacy of Citicoline as a Neuroprotector in children with post cardiac arrest: a randomized controlled clinical trial | 2021 | Salamah, A.,et al | 2 |
| Functional connectivity and upper limb function in patients after pediatric arterial ischemic stroke with contralateral corticospinal tract wiring | 2021 | Steiner, L.,et al | 3 |
| Endovascular Treatment for Acute Ischemic Stroke in Children: Experience From the MR CLEAN Registry | 2021 | van Es, A.,et al | 2 |
| Feasibility and safety of intranasally administered mesenchymal stromal cells after perinatal arterial ischaemic stroke in the Netherlands (PASSIoN): a first-in-human, open-label intervention study | 2022 | Baak, L. M.,et al | 2 |
| Early, Intensive, Lower Extremity Rehabilitation Shows Preliminary Efficacy After Perinatal Stroke: Results of a Pilot Randomized Controlled Trial | 2022 | Hurd, C.,et al | 3 |
| Association Between Thrombectomy and Functional Outcomes in Pediatric Patients With Acute Ischemic Stroke From Large Vessel Occlusion | 2023 | Bhatia, K. D.,et al | 2 |
| Intravenous thrombolysis for treatment of pediatric acute ischemic stroke: Analysis of 20 years of population-level data in the United States | 2023 | Dicpinigaitis, A. J.,et al | 2 |
| Hypercapnic hypoxia as a rehabilitation method for patients after ischemic stroke | 2024 | Alekseeva, T. M.,et al | 3 |
| Hydroxyurea to prevent brain injury in children with sickle cell disease (HU Prevent)-A randomized, placebo-controlled phase II feasibility/pilot study | 2024 | Casella, J. F.,et al | 4 |
| Detailed statistical analysis plan for ALBINO: effect of Allopurinol in addition to hypothermia for hypoxic-ischemic Brain Injury on Neurocognitive Outcome - a blinded randomized placebo-controlled parallel group multicenter trial for superiority (phase III) | 2024 | Engel, C.,et al | 2 |
| Remote ischemic conditioning prevents ischemic cerebrovascular events in children with moyamoya disease: a randomized controlled trial | 2024 | Huang, S. F.,et al | 4 |
| A population pharmacokinetics model of balovaptan to support dose selection in adult and pediatric populations | 2024 | Schaedeli Stark, F.,et al | 2 |
| Endovascular thrombectomy for childhood stroke (Save ChildS Pro): an international, multicentre, prospective registry study | 2024 | Sporns, P. B.,et al | 2 |
| Determinants of Timely Access to Recanalization Treatments and Outcomes in Pediatric Ischemic Stroke | 2024 | Tudorache, R.,et al | 2 |

**Classifications--Class 1:** Detection and diagnosis of pediatric IS; **Class 2:** Therapy and efficacy of pediatric IS; **Class 3:** Recovery and outcomes of pediatric IS; **Class 4:** Etiology and risk factors of pediatric IS.
